# The promise of record linkage for assessing the uptake of health services in resource constrained settings: a pilot study from South Africa

**DOI:** 10.1186/1471-2288-14-71

**Published:** 2014-05-24

**Authors:** Chodziwadziwa W Kabudula, Benjamin D Clark, Francesc Xavier Gómez-Olivé, Stephen Tollman, Jane Menken, Georges Reniers

**Affiliations:** 1 MRC/Wits Rural Public Health and Health Transitions Research Unit (Agincourt), School of Public Health, Faculty of Health Sciences, University of the Witwatersrand, Johannesburg, South Africa; 2 Department of Ecology, Evolution and Environmental Biology, Columbia University, New York, USA; 3 Umeå Centre for Global Health Research, Division of Epidemiology and Global Health, Department of Public Health and Clinical Medicine, Umeå University, Umeå, Sweden; 4 INDEPTH Network, Accra, Ghana; 5 Institute of Behavioral Science, University of Colorado, Boulder, Colorado, USA; 6 Department of Population Health, London School of Hygiene and Tropical Medicine, London, UK

**Keywords:** Health and Demographic Surveillance System (HDSS), Record linkage, Health facilities, South Africa, Population surveillance

## Abstract

**Background:**

Health and Demographic Surveillance Systems (HDSS) have been instrumental in advancing population and health research in low- and middle- income countries where vital registration systems are often weak. However, the utility of HDSS would be enhanced if their databases could be linked with those of local health facilities. We assess the feasibility of record linkage in rural South Africa using data from the Agincourt HDSS and a local health facility.

**Methods:**

Using a gold standard dataset of 623 record pairs matched by means of fingerprints, we evaluate twenty record linkage scenarios (involving different identifiers, string comparison techniques and with and without clerical review) based on the Fellegi-Sunter probabilistic record linkage model. Matching rates and quality are measured by their sensitivity and positive predictive value (PPV). Background characteristics of matched and unmatched cases are compared to assess systematic bias in the resulting record-linked dataset.

**Results:**

A hybrid approach of deterministic followed by probabilistic record linkage, and scenarios that use an extended set of identifiers including another household member’s first name yield the best results. The best fully automated record linkage scenario has a sensitivity of 83.6% and PPV of 95.1%. The sensitivity and PPV increase to 84.3% and 96.9%, respectively, when clerical review is undertaken on 10% of the record pairs. The likelihood of being linked is significantly lower for females, non-South Africans and the elderly.

**Conclusion:**

Using records matched by means of fingerprints as the gold standard, we have demonstrated the feasibility of fully automated probabilistic record linkage using identifiers that are routinely collected in health facilities in South Africa. Our study also shows that matching statistics can be improved if other identifiers (e.g., another household member’s first name) are added to the set of matching variables, and, to a lesser extent, with clerical review. Matching success is, however, correlated with background characteristics that are indicative of the instability of personal attributes over time (e.g., surname in the case of women) or with misreporting (e.g., age).

## Background

Health and Demographic Surveillance Systems (HDSS) enumerate populations in geographically well-defined areas and prospectively collect detailed information on vital events including births, deaths, and migrations, as well as complementary data covering health, social and economic indicators [1-3]. These data allow for population-based investigations of population and health dynamics and their determinants in low- and middle- income countries where vital registration systems are often weak [2]. However, the scope of analysis possible with datasets from most HDSSs is constrained by the lack of integration with other administrative data, including those emanating from health facilities. For example, HDSS data have demonstrated reductions in overall mortality levels in HIV/AIDS affected African populations following the expansion of antiretroviral therapy programs [4-6], but residual AIDS mortality remains important. In order to achieve further reductions in mortality levels, it is important to understand whether individuals dying of AIDS have had any contact with the health facilities and the nature of that contact (e.g., diagnosis, in care awaiting treatment initiation, on first line treatment). Unfortunately, this is difficult without linking HDSS and health facility data. The best measures currently available on health care utilization rely on retrospective reports from living patients or from relatives or caretakers of the deceased. Data from health facilities alone do not address these types of research and policy questions either as they fail to account for individuals who never make contact with the health facility.

Record linkage of electronic patient records based on conventional personal identifiers is a cost-effective means for integrating information from different sources [7]. This approach has been applied extensively to generate datasets for epidemiological studies in higher income settings (e.g., United States of America [8,9], Wales [10], Australia [11-13], Italy [14], Canada [15], Netherlands [16] and the United Kingdom [17]) but it is much less common in African populations or in the context of HDSS^a^. Obstacles to record linkage in these settings include the lack of unique and ubiquitous identification systems (e.g., national insurance or social security number), variation in the transcription of names, imprecision in the reporting of dates, and other data quality related issues.

In this study, we assess the feasibility of record linkage with conventional personal identifiers (e.g., name, age, address) between an HDSS and a health facility in South Africa using data from the Agincourt HDSS and patient attendance records from a local government health facility. Our study is unusual because we first construct a gold standard dataset of records matched by means of fingerprints and subsequently use it to assess the coverage and accuracy of various record linkage scenarios. Finally, we compare the background characteristics of matched and unmatched cases, and evaluate compositional differences in the linked and full dataset.

There are three reasons why we pursue record linkage on conventional personal identifiers as opposed to record linkage on fingerprints. First, fingerprints are known to have a very high specificity but relatively low sensitivity [18]. This property renders fingerprint-matched records a good gold standard for evaluating other record linkage approaches, but makes it less desirable as a record linkage solution itself. Other biometric identifiers (e.g., iris scan and facial recognition) may outperform fingerprints in that regard. Second, record linkage on the basis of fingerprints (or any other biometric) would require the HDSS to collect and store fingerprints for all its residents, and we chose to assess the utility of a cheaper method. Third, fingerprint-based record linkage would require that fingerprint collection becomes part of the patient administration systems in all health facilities. Since many health facilities in low- and middle- income countries do not have computerized health management information systems, this is unlikely to become a realistic solution in the short term.

## Methods

### Datasets

Three datasets are used in this study. The first dataset (dataset1) consists of identifiers of 93,507 individuals who were under surveillance by the Agincourt HDSS at any time between 1 August 2009 and 1 August 2010. The Agincourt HDSS encompasses 27 villages spread over 420 km^2^ of semi-arid scrubland in rural northeast South Africa in the Bushbuckridge sub-district of Ehlanzeni district, Mpumalanga Province [19,20]. The population under surveillance is largely Xitsonga-speaking with one-third being former Mozambican refugees who arrived in the 1980s- and their descendants.

The second dataset (dataset2) consists of identifiers and fingerprints of 2,865 individuals aged 18 years and above from two villages in the Agincourt HDSS. The fingerprints were collected during a mini-census in which 6,185 residents aged 18 years and above were visited in their homes between November 2008 and April 2009. Verbal informed consent was obtained to collect fingerprints and to link the Agincourt HDSS database record to any visits to Agincourt Health Centre (AHC), which is one of eight local health facilities within the Agincourt HDSS. Between two and four fingerprints were collected from each individual who agreed to participate in the study. A large number of the individuals from whom fingerprints could not be collected were absent during the household visits (circular labor migration is very common in the area). Among the individuals who were found at home (2,965 individuals), only 45 individuals refused participation, and technical problems with the collection of fingerprints (often due to scars or cuts on the finger) accounted for 55 cases. Details about the community-based fingerprint collection are presented elsewhere [21].

The third dataset (dataset3) consists of identifiers and fingerprints that were collected as part of a pilot electronic patient registration system at the reception desk of the AHC. This electronic patient registration system was managed by SAP Meraka Unit for Technology Development (UTD) and the School of Public Health from the University of the Witwatersrand [22]. The data were collected between August 2008 and August 2010. Identifiers were collected from 10,790 individuals and fingerprints from 3,633 of them. At least two fingerprints were collected from 93.6% of these 3,633 individuals. Fingerprints were not collected for extended periods of time at the AHC because of technical problems that the personnel at the reception desk could not independently resolve.

Identifiers included in dataset3 are those that are routinely collected at the AHC such as first name, surname, sex, date of birth, and place of residence, and attributes that we added to the patient registration for the purpose of this study (e.g., the first and surname of another household member). National ID number and telephone number were also on the list of identifiers to be collected but were not consistently reported by individuals attending the AHC. In anticipation of this (and future) record linkage studies we collect National ID number and telephone number(s) during the annual Agincourt HDSS census update since 2007 and 2011 respectively. Additionally, we have included the collection of *other names* for all individuals in the annual Agincourt HDSS census update since 2011.

### Gold standard dataset

We constructed a dataset of matched individuals from the Agincourt HDSS and the AHC by linking individuals’ fingerprints in dataset2 with the fingerprints in dataset3. Matching of the fingerprints was performed using the SAGEM MorphoSmart Compact Biometric Module (CBM) with a threshold of 5 as recommended by the manufacturer [23]. The threshold can be varied from 0 to 10 with higher thresholds producing less false positive cases and lower thresholds producing fewer false negatives. The threshold of 5 has a false acceptance rate (FAR = 1-Specificity) <0.01% [23].

The matching of fingerprints from the 2,865 individuals in the two target villages of the Agincourt HDSS with those captured from the 3,633 individuals that visited the AHC resulted in 623 matched record pairs. At least two fingerprints were matched in 393 (63.08%) cases.

### Record linkage with conventional personal identifiers

We use two approaches for linking individuals in dataset1 with individuals in dataset3. In the first approach we exclusively use probabilistic record linkage methods. In the second approach we use a hybrid strategy whereby we first link records deterministically and thereafter match the remaining records using probabilistic methods. Deterministic record linkage designates a pair of records from two data sources as belonging to the same individual when they match on a unique identifier such as fingerprints, a social security or national identification number, or a set of conventional personal identifiers (e.g., the combination of first name, last name and date of birth) [24-27]. Probabilistic record linkage classifies a pair of records from two data sources as belonging to the same individual based on the statistical probability that common identifiers drawn from the two data sources belong to the same individual [28-33]. Whereas deterministic linkage is most suitable when unique identifiers are available and the quality of the data are high, probabilistic linkage yields better results when unique identifiers are lacking or in situations where there is variation in reporting or transcription of personal identifiers [24,29,34-36].

We first define 15 probabilistic record linkage scenarios (S1-S15) based on different combinations of personal identifiers or linking variables (first name, surname, day of birth, month of birth, year of birth, village and first name and surname of another household member), and various string comparison techniques to accommodate typographical errors and spelling variation in first and surnames. The string comparison techniques used are the Jaro-Winkler (JW) string comparator [37], the Soundex phonetic encoding and the Double Metaphone phonetic encoding [38]. Details about these probabilistic linkage scenarios are given in Table 1.

**Table 1:** Linkage scenarios by identifiers and string comparison techniques applied to names

		**String comparison techniques applied to first and surnames**					
		**Exact**	**JW ≥ 0.7**	**JW ≥ 0.9**	**DM**	**Soundex**	**JW ≥ 0.9 or DM or soundex**
Identifiers used	Routinely collected identifiers*	S1	S2	S3	S4	S5	S6
	Routinely collected identifiers + household member first name	S7	S8	S9	S10	S11	S12
	Routinely collected identifiers + household member first name and surname			S13	S14	S15	
	Deterministic linkage on National ID Number or telephone number followed by best of S1-S15**						S16
	S16 + clerical review of 5%, 10%, 15%, and 20% of record pairs above and below the threshold value above which record pairs are automatically accepted as matches						S17-S20

*Routinely collected identifiers = first name, last name, sex, day of birth, month of birth, year of birth and village; JW = Jaro-Winkler; DM = double metaphone code.

**The best of the 15 probabilistic linkage scenarios is the one that yields the maximum sensitivity and PPV.

Thereafter, we create another scenario (S16 in Table 1), which first matches records deterministically using National ID number or a combination of telephone number and first name, and subsequently matches the remaining cases using the scenario that yields the maximum sensitivity and positive predictive value (PPV) among the first 15 probabilistic linkage scenarios.

Since the number of possible record pair comparisons in two data files to be linked is enormous - equal to the product of the number of records on each file (over 1 billion record pairs in our case) - we use a technique called “blocking” to restrict the comparison space to blocks or pockets of record pairs where one or more variables match exactly [31]. Blocking is useful for reducing computing time, but may decrease the sensitivity if blocking variables are measured with error. In order to minimize the effect of errors in blocking variables, we use three blocking schemes: exact match on sex and year of birth (BS-1), exact match on sex and village (BS-2) and exact match on the first letter of the first name and surname and age difference of not more than 10 years (BS-3). We combine linked record pairs from the different blocks and extract a unique set of linked record pairs as a combination of all distinct record pairs and the record pair with the highest matching score (see below) in cases where a record from dataset3 is matched to multiple records in dataset1.

A key step in probabilistic linkage is the estimation of weights to indicate the contribution of each identifier to the probability of accurately designating a pair of records from two different sources as either a match or non-match [27,30,31]. For each common identifier, *i*_,_ available in the two data sources, the process involves first estimating the probability that the identifier agrees given that the two records belong to the same individual, denoted by *m*_*i*_, and the probability that the identifier agrees given that the two records do not belong to the same individual, denoted by *u*_*i*_[30,31,33]. The *m*_*i*_ values depend on measurement and reporting error in an identifier whereas the *u*_*i*_ values depend on the number of distinct values of an identifier and their frequencies [32,39]. Identifiers collected and recorded with good quality in both datasets have higher *m*_*i*_ values. On the other hand, identifiers with many different values are less likely to agree by chance, and hence, have lower *u*_*i*_ values. In record pairs where identifier *i* agrees, the identifier is assigned a weight value of $\log_{2}\frac{m_{i}}{u_{i}}$ and where identifier *i* disagrees a weight value of $\log_{2}\frac{1-m_{i}}{1-u_{i}}$ is assigned. Thereafter each record pair is classified as a match or non-match depending on whether the sum of the weights on all the identifiers used (matching score) is above or below a threshold value above which record pairs are automatically accepted as matches.

For each scenario, we estimate *m*_*i*_ and *u*_*i*_ probabilities from the datasets to be linked using an Expectation Maximization (EM) algorithm [31,40,41] based on the Fellegi-Sunter model [42]. Following Méray et al. [39] and Tromp et al. [43], we use an estimate of the proportion of true matches among all possible record pair combinations to determine a scenario-specific threshold matching score above which record pairs are automatically accepted as matches.

Finally, we create four more scenarios (S17-S20 in Table 1) that use scenario S16 as the starting point and add clerical review for a selection of record pairs immediately above and below the threshold value. These scenarios allocate 5% (S17), 10% (S18), 15% (S19) and 20% (S20) of record pairs immediately above and below the threshold value in scenario S16 to clerical review. Two reviewers independently review the targeted record pairs and classify each of them as a match or non-match. When the two reviewers disagree, a third reviewer adjudicates over the match status.

There are four possible outcomes from record linkage: true matches (true positives), true non-matches (true negatives), mismatches (false positives) and false non-matches (false negatives) [44]. Coverage and accuracy of each linkage scenario can thus be assessed by four indices: sensitivity, specificity, PPV and negative predictive value (NPV). Sensitivity is the proportion of true matches that are produced by the linkage algorithm, specificity is the proportion of true non-matches, PPV is the proportion of matches produced by the linkage algorithm that are true matches and NPV is the proportion of non-matches produced by the linkage algorithm that are true non-matches [45]. However, as the number of true non-matches are often very large, specificity and NPV are not very informative [34]. Therefore, we report sensitivity and PPV for each linkage scenario against the gold standard.

### Bias in the record-linked dataset

Because record linkage may produce mismatches and missed matches it is recommended that linked and unlinked records are assessed for systematic bias [46,47]. We thus select cases for which we know the true match status from the gold standard dataset and regress the record linkage outcome on individual characteristics using a logistic model. Age, sex, residency status in the Agincourt HDSS, nationality, level of education, employment status and household wealth quintile are considered as predictors of accurate linkage. Wealth quintiles are derived from data on ownership of assets such as cattle, car, and cell phone as well as access to amenities including drinking water and sanitation using principal components analysis [48]. In addition to this individual-level assessment of factors associated with linkage success, we also compare the distribution of background characteristics in the gold standard and record linked datasets using Pearson Chi squared tests.

### Implementation

We implemented the record linkage with conventional personal identifiers in Microsoft SQL Server 2008. The EM algorithm used to estimate the *m* and *u* probabilities and the proportion of true matches among all possible record pair combinations is implemented in Microsoft C# and integrated into Microsoft SQL Server as a common language runtime (CLR) function. The Soundex algorithm is a Microsoft SQL Server built-in function. The JW and Double Metaphone algorithms were integrated into Microsoft SQL Server as CLR functions. The JW algorithm is part of the SimMetrics library and its source code is freely available [49]. The source code for the Double Metaphone algorithm is also freely available [50]. Data analysis is conducted in Stata version 12.

### Ethical approval

The study received ethical approvals from the University of the Witwatersrand Human Research Ethics Committee (Clearance number: M071141) and the Mpumalanga Provincial Department of Health Research and Ethics Committee.

## Results

**Figure 1:**
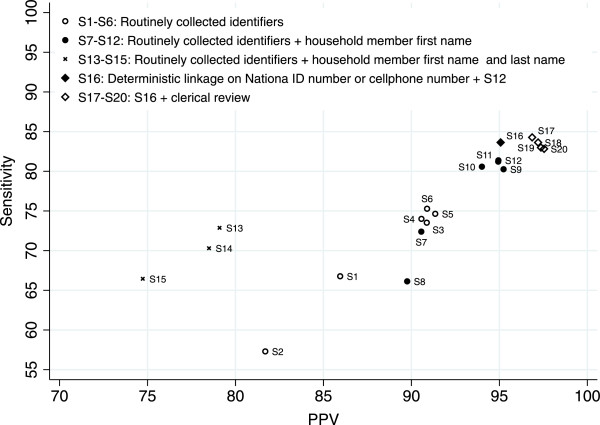
**Sensitivity and positive predictive values (PPVs) in various linkage scenarios.** See Table 1 for a description of the scenarios.

The level of completeness of the identifiers used as linking variables in the various scenarios is higher in the data from the Agincourt HDSS compared to that from the AHC (Table 2). Village, another household member’s first and surname, National ID number and telephone number are often missing in the AHC dataset. None of these characteristics are routinely recorded in health facilities.Figure 1 plots the sensitivity against PPV for each of the record linkage scenarios. Scenarios solely based on identifiers that are routinely collected in health facilities (S1-S6) have sensitivity ranging from 57.30% to 74.64%, and PPV ranging from 81.69% to 91.72%. Adding another household member’s first name to the set of matching variables (S7-S12) considerably improved sensitivity (range: 66.13% to 81.35%) and PPV (range: 89.76% to 94.94%). However, adding another household member’s last name (S13-S15) to the set of identifying variables leads to deterioration in the matching rates and accuracy. The string comparison methods that produce the best results are the JW with a threshold value of 0.9, the Double Metaphone and Soundex. Differences between these three are small. Scenarios where we consider an exact match on names or a JW score above 0.7 have a markedly lower sensitivity and PPV.

**Table 2:** Completeness of identifiers from both sources

**Identifier**	**Percentage of individuals with complete information**	
	**From Agincourt HDSS (*n*** **= 93 507)**	**From Agincourt Health Centre (*n*** **= 10790)**
First name	100.00	100.00
Surname	100.00	100.00
Other first name	35.57	6.14
Sex	100.00	99.95
Date of birth	100.00	100.00
Village	100.00	81.17
Household member first name	98.48	77.29
Household member surname	98.48	76.60
ID number	67.14	1.55
Telephone number	37.48	26.67

With sensitivity of 81.38% and PPV of 94.94%, scenario S12 produces the best results among the purely probabilistic linkage scenarios. Matching statistics further improve by first matching records deterministically using National ID number or telephone number and first name, and subsequently matching the remaining records with probabilistic methods using the criteria set forth in scenario S12. This hybrid record linkage approach (S16) increases sensitivity to 83.63% and PPV to 95.07%. The improvement in matching statistics is only marginal, however, and probably due to the fact that these attributes have a substantial number of missing values in either one or both datasets.

The inclusion of clerical review in the linkage process results in modest improvements in PPV. Allocating 5% of the record pairs below and above the threshold value in scenario S16 to clerical review (S17) yields the best results in terms of maximizing both sensitivity (84.27%) and PPV (96.86%). The other scenarios involving clerical review produce small gains in PPV, but are considerably more labour intensive. For example, for scenario S17, 1131 record pairs were reviewed and it took the two reviewers an average of 5 hours each to complete the task whereas for scenario S20, 3492 record pairs were reviewed, which required an average of 15 hours per reviewer.

In Table 3, we present a number of background characteristics of individuals and their association with matching success. The records come from the gold standard dataset in which record pairs are matched using fingerprints, and match success in record linkage scenarios based on conventional personal identifiers is the outcome of interest. This analysis is conducted for three of the scenarios defined in Table 1: (i) the best fully automated scenario that uses only personal identifiers that are routinely collected in health facilities (S6), (ii) the best fully automated record linkage scenario based on an extended set of personal identifiers and wherein deterministic and probabilistic linkage methods are combined (S16), and (iii) S17, which is equivalent to S16 with the addition of clerical review of 5% of the record pairs with a matching score immediately above and below the threshold value.

**Table 3:** Background characteristics associated with successful matching in the dataset of records matched by means of fingerprints

**Variable**		**n**	**Linkage scenario 6**		**Linkage scenario 16**		**Linkage scenario 17**	
			**Matched**	**Multivariable**	**Matched**	**Multivariable**	**Matched**	**Multivariable**
			***n *** **(%)**	**OR (95% CI)**	***n *** **(%)**	**OR (95% CI)**	***n *** **(%)**	**OR (95% CI)**
		623	492 (79.0)		551 (88.4)		552 (88.6)	
**Sex**								
	Female	511	395 (77.3)	1	445 (87.1)	1	447 (87.5)	1
	Male	112	97 (86.6)	2.86 (1.41-5.82)*	106 (94.6)	4.38 (1.52-12.61)*	105 (93.8)	3.34 (1.25-8.97)*
**Age**								
	18-34	334	284 (85.0)	1	308 (92.2)	1	308 (92.2)	1
	35-49	125	100 (80.0)	0.99 (0.53-1.84)	112 (89.6)	0.84 (0.36-1.93)	115 (92.0)	1.21 (0.5-2.92)
	50-64	89	66 (74.2)	0.76 (0.35-1.66)	78 (87.6)	0.75 (0.27-2.14)	77 (86.5)	0.75 (0.27-2.12)
	65+	75	42 (56.0)	0.35 (0.15-0.85)*	53 (70.7)	0.21 (0.07-0.63)*	52 (69.3)	0.25 (0.08-0.74)*
**Ethnicity**								
	Other	96	67 (70.0)	1	76 (79.2)	1	75 (78.1)	1
	South African	527	425 (80.7)	1.3 (0.71-2.37)	475 (90.1)	1.82 (0.88-3.77)	477 (90.5)	2.1 (1.02-4.33)*
**Residence status**								
	Permanent	574	450 (78.4)	1	506 (88.1)	1	507 (88.3)	1
	Temporary and other	49	42 (85.7)	1.63 (0.54-4.88)	45 (91.8)	1.28 (0.28-5.89)	45 (91.8)	1.4 (0.31-6.44)
**Highest level of education**								
	None	97	54 (55.7)	1	71 (73.2)	1	69 (71.1)	1
	Some primary	191	144 (75.4)	1.46 (0.76-2.83)	164 (85.8)	1.16 (0.51-2.63)	166 (87.0)	1.43 (0.64-3.22)
	Post primary	302	267 (88.4)	2.73 (1.18-6.36)*	288 (95.4)	2.62 (0.87-7.92)	288 (95.4)	3.05 (1.01-9.24)*
**Employment**								
	Not working	514	413 (80.4)	1	462 (89.8)	1	460 (89.5)	1
	Working	93	70 (75.3)	0.68 (0.37-1.25)	79 (85.0)	0.53 (0.25-1.14)	81 (87.1)	0.71 (0.32-1.58)
**Wealth quintile**								
	Lowest	44	28 (63.6)	1	33 (75.0)	1	34 (77.3)	1
	Second	84	62 (73.8)	1.48 (0.63-3.49)	75 (89.3)	2.42 (0.84-6.98)	73 (90.0)	1.63 (0.57-4.62)
	Middle	125	100 (80.0)	1.89 (0.82-4.37)	108 (86.4)	1.60 (0.6-4.25)	110 (88.0)	1.58 (0.58-4.36)
	Fourth	172	136 (79.1)	1.81 (0.8-4.11)	152 (88.3)	2.08 (0.78-5.54)	150 (87.2)	1.47 (0.55-3.93)
	Highest	184	159 (86.4)	2.9 (1.24-6.75)*	174 (94.5)	4.4 (1.51-12.84)*	175 (95.1)	4.03 (1.34-12.17)*
**Goodness-of-fit**								
	Pseudo R^2^, Wald *χ*^2^ (*p*-value)		0.11, 56.89 (<0.0001)		0.16, 51.94 (<0.0001)		0.16, 53.76 (<0.0001)	

Statistical significance: * = *p*-value < 0.05.

Background characteristics associated with a lower matching likelihood in a multivariable model are female gender, old age, and low socioeconomic status (being below the highest wealth quintile). The coefficients for age indicate that matching rates deteriorate above age 50 (significantly above age 65), which suggests that reporting of personal identifiers in older respondents may not be as reliable. Being non South African is associated with lower matching success only in scenario S17 whereas having received less than primary education is associated with lower matching success in both scenarios S6 and S17. Interestingly, the scenarios that produce the best matching statistics (S16 and S17) do not necessarily produce samples of matched records that are less biased (i.e., significant predictors of matching success are similar across the three scenarios in Table 3).

Although matched and non-matched records differ in terms of some of their background characteristics, the distribution of background characteristics in the fingerprint linked dataset and the dataset generated via record linkage on conventional personal identifiers is quite similar for all the three scenarios considered here (Table 4). The reason is that the algorithms will select an individual with similar personal attributes (gender, age, etc.), even if it is not an exact match.

**Table 4:** Distribution of background characteristics in the dataset matched by means of fingerprints compared to three datasets of records matched using conventional personal identifiers

**Variable**		**Matched on fingerprint (n = 623)**	**Matched with scenario 6 (n = 492)**		**Matched with scenario 16 (n = 551)**		**Matched with scenario 17 (n = 552)**	
		***n *** **(%)**	***n *** **(%)**	*p*-value^*^	***n *** **(%)**	*p*-value^*^	***n *** **(%)**	*p*-value^*^
**Sex**								
	Female	511 (82.0)	395 (80.3)		445 (80.8)		447 (81.0)	
	Male	112 (18.0)	97 (19.7)	0.460	106 (19.2)	0.579	105 (19.0)	0.645
**Age**								
	18-34	334 (53.6)	284 (57.7)		308 (55.9)		308 (55.8)	
	35-49	125 (20.1)	100 (20.3		112 (20.3)		115 (20.8)	
	50-64	89 (14.3)	66 (13.4)		78 (14.2)		77 (14.0)	
	65+	75 (12.0)	42 (8.5)	0.240	53 (9.6)	0.601	52 (9.4)	0.528
**Ethnicity**								
	Other	96 (15.4)	67 (13.6)		76 (13.8)		75 (13.6)	
	South African	527 (84.6)	425 (86.4)	0.401	475 (86.2)	0.434	477 (86.4)	0.377
**Residence status**								
	Permanent	574 (92.1)	450 (91.5)		506 (91.8)		507 (91.8)	
	Temporary and other	48 (7.7)	42 (8.5)	0.595	45 (8.2)	0.617	45 (8.2)	0.618
**Highest level of education**								
	None	97 (15.6)	54 (11.0)		71 (12.9)		69 (12.5)	
	Some primary	191 (30.7)	144 (29.3)		164 (29.8)		166 (30.1)	
	Post primary	302 (48.5)	267 (54.3)	0.098	288 (52.3)	0.491	288 (52.2)	0.426
**Employment**								
	Not working	514 (82.5)	413 (83.9)		462 (83.4)		460 (83.3)	
	Working	93 (14.9)	70 (14.2)	0.660	79 (14.3)	0.643	81 (14.7)	0.795
**Wealth quintile**								
	Lowest	44 (7.1)	28 (5.7)		33 (6.0)		34 (16.2)	
	Second	84 (13.5)	62 (12.6)		75 (13.6)		73 (13.2)	
	Middle	125 (20.1)	100 (20.3)		108 (19.6)		110 (19.9)	
	Fourth	172 (27.6)	136 (27.6)		152 (27.6)		150 (21.2)	
	Highest	184 (29.5)	159 (32.3)	0.753	174 (31.58)	0.912	175 (31.7)	0.952

*p-value using chi-squared test computed separately for records in each scenario compared to records matched by means of fingerprints.

## Discussion

We have evaluated the coverage and quality of record linkage in rural South Africa between the Agincourt HDSS and patient administration records from a health facility in its vicinity. We created a gold standard dataset of records matched by means of fingerprints and use it to evaluate the performance of 20 record linkage scenarios with conventional personal identifiers. The various record linkage scenarios can be distinguished by four attributes. First, one set of scenarios uses only personal identifiers that are routinely collected in health facilities (first name, surname, date of birth, sex and village) whereas another set of scenarios uses an extended set of identifiers (adding another household member’s names, national ID number and telephone number). Second, some scenarios use purely probabilistic methods of record linkage, whereas others follow a hybrid approach where records are first matched deterministically using National ID number or telephone number and first name, and the remainder are retained for probabilistic record linkage. Third, we use different string comparison metrics for names. Finally, we define purely automated record linkage scenarios as well as scenarios involving clerical review of a subset of record pairs.

Record linkage scenarios with the most satisfying results are those that follow a hybrid approach of deterministic followed by probabilistic record linkage, and those that use an extended set of identifiers including another household member’s first name, National ID number and telephone number. Worth noting is that another household member’s first name is a substantially better matching variable than his or her surname as the latter is often the same as that of the person to be linked and does not add much new information. In terms of string comparison metrics, the best results are obtained in scenarios that use a combination of Soundex, Double Metaphone and a Jaro-Winkler score above 0.9 (see also [51]).

Fully automated record linkage based on a set of personal identifiers that are routinely collected at health facilities (S6 in Table 1) has a sensitivity of 75.28% and PPV of 90.89%. The best fully automated record linkage scenario based on an extended set of identifiers and following a hybrid deterministic-probabilistic approach (S16), yields a sensitivity of 83.63% and PPV of 95.07%. The sensitivity and PPV increase to 84.27% and 96.86%, respectively, when clerical review is performed on 10% of the record pairs around the matching score threshold of scenario S16. Even though these results are very encouraging, it is likely that they could be improved further by more comprehensive collection of National ID numbers and telephone numbers in both the Agincourt HDSS and the health facility.

Matching rates are significantly worse for women (compared to men), for former Mozambican refugees (compared to native South Africans), and for the poorly educated and older respondents. The association between these background characteristics and matching rates is similar in all record linkage scenarios, irrespective of their sensitivity and PPV. The lower matching success for women may be because some of them change names upon marriage and may be known by their husband’s name in one data source and registered under their maiden name in another data source. As for older respondents, the lower matching success could be a result of poorer reporting with age or an effect of older generations not having accurate information on some of their identifiers such as date of birth. The lower matching success for Mozambicans could be related to their legal status, but we have no means of verifying this. These analyses of the individual-level correspondence in matching success are thus indicative of systematic bias in all of the record linkage scenarios considered here. It is also worth noting, however, that the distributions of socio-demographic background characteristics in the gold standard and record-linked datasets are very similar, which suggests that record-linked datasets may still be used for assessing equitable uptake of services.

## Conclusion

Using records matched by means of fingerprints as the gold standard, we have demonstrated the feasibility of fully automated probabilistic record linkage using identifiers that are routinely collected in health facilities in South Africa. Our study also shows that matching statistics can be improved if other identifiers (e.g., another household member’s first name) are added to the set of matching variables, and, to a lesser extent, with clerical review. Matching success is, however, correlated with background characteristics that are indicative of the instability of personal attributes over time (e.g., surname in the case of women) or with misreporting of attributes (e.g., age).

## Endnotes

^a^Some HDSS that have been built around a health facility or manage a health facility as part of their research operation (e.g., the Kilifi HDSS or the Masaka HDSS). In these studies, the data systems are well integrated.

## Competing interests

The authors declare that they have no competing interests.

## Authors’ contributions

CWK and GR designed and executed the study, conducted the analyses, and wrote the first draft of the manuscript. BDC and FXGO provided assistance with programming and the organization of the fieldwork. All co-authors made substantial contributions to the study design and manuscript preparation. All authors approved the final version of the manuscript.

## Pre-publication history

The pre-publication history for this paper can be accessed here:

http://www.biomedcentral.com/1471-2288/14/71/prepub
